# Education in the genomics era: Generating high-quality genome assemblies in university courses

**DOI:** 10.1093/gigascience/giaa058

**Published:** 2020-06-03

**Authors:** Stefan Prost, Sven Winter, Jordi De Raad, Raphael T F Coimbra, Magnus Wolf, Maria A Nilsson, Malte Petersen, Deepak K Gupta, Tilman Schell, Fritjof Lammers, Axel Janke

**Affiliations:** 1 LOEWE-Centre for Translational Biodiversity Genomics, Senckenberganlage 25, 60325 Frankfurt, Germany; 2 South African National Biodiversity Institute, National Zoological Garden, 232 Boom Street, Pretoria 0001, South Africa; 3 Institute for Ecology, Evolution and Diversity, Goethe University, Max-von-Laue Strasse 13, 60438 Frankfurt, Germany; 4 Senckenberg Biodiversity and Climate Research Centre, Senckenberganlage 25, 60325 Frankfurt, Germany

**Keywords:** genome assembly, MinION, Oxford Nanopore Technologies, teaching, university education

## Abstract

Recent advances in genome sequencing technologies have simplified the generation of genome data and reduced the costs for genome assemblies, even for complex genomes like those of vertebrates. More practically oriented genomic courses can prepare university students for the increasing importance of genomic data used in biological and medical research. Low-cost third-generation sequencing technology, along with publicly available data, can be used to teach students how to process genomic data, assemble full chromosome-level genomes, and publish the results in peer-reviewed journals, or preprint servers. Here we outline experiences gained from 2 master's-level courses and discuss practical considerations for teaching hands-on genome assembly courses.

## Background

The number of published genome assemblies has increased exponentially since the publication of the human genome in 2001 [1]. Back then, large international consortia and vast amounts of funding were required to complete this task. Today even small research groups can generate high-quality genome assemblies up to full chromosome level. An important step in this progression was the advent of “third-generation” sequencing with the release of Pacific Biosciences’ and Oxford Nanopore Technologies’ sequencing platforms. These technologies perform real-time sequencing of long, single DNA molecules and require no amplification to increase the sequencing detection signal strength [2]. The inclusion of these technologies has drastically improved the quality of generated genome assemblies and substantially decreased costs, thus enabling even small research groups to realise high-quality genome assemblies. The ongoing “genomic revolution” has increased the need for practical university courses on genome assembly and genomics in general.

One of these third-generation sequencing platforms is Oxford Nanopore Technologies' MinION. It is a USB drive–sized sequencer that has gained in popularity over the past 5 years owing to its potential to generate very long reads, its relatively low cost, and its portability [3, 4]. Sequencing is performed by measuring ionic current changes when a single-stranded DNA molecule passes through a “nanopore” in the device's biological membrane [5]. Its portability, ease of use, and relatively low cost make it an ideal and effective teaching tool in classroom settings [6, 7], as well as in the field [8]. Studies on the educational use of the MinION device have mainly focused on methods such as DNA- or metabarcoding, or genome sequencing of bacteria or bacteriophages. These approaches have the advantage that they are easy to conduct, do not require large servers for the data processing, and are relatively inexpensive. However, today, the combination of (i) nanopore-based sequencing along with (ii) new efficient bioinformatic assembly pipelines and (iii) (publicly available) short-read data offer the possibility of generating chromosome-level assemblies, even of complex vertebrate genomes, as part of university courses.

Here we report our experience and provide practical aspects of teaching hands-on vertebrate genome assembly courses at the master's level.

## Practical Courses in the Genomics Era

Over the past 2 years, we have taught 2 master's level courses, each 6 weeks long, focusing on the assembly and analysis of vertebrate genomes, and the required theoretical and biological background. In these courses, students gained practical experience in extracting high molecular weight DNA (hmwDNA) and preparing sequencing libraries and subsequently sequenced the genomic DNA on the MinION device in the first part of the course (see Table 1 and Supplementary Material 1). These data, in combination with either publicly available or previously generated short-read data, were then used to generate highly continuous vertebrate genome assemblies in the second part of the course (see Table 1 and Supplementary Material 1). The inclusion of dedicated sessions on the basics of laboratory work and bioinformatics enables the active participation of students from a variety of disciplines (such as biology or bioinformatics) even without prior knowledge in these fields. We recommend keeping the number of students <15 to enable direct interactions. This is important especially for students who have no or little bioinformatics or laboratory experience and might otherwise struggle to keep up with the course.

**Table 1:** Example of a course outline

**Part 1—Laboratory Processing and Basic Training**	
Week 1	Theory: Introduction to genome sequencing techniques and analyses
	Hands-on: Laboratory work (laboratory safety guidelines, DNA isolation, quality assessment, nanopore library preparation, sequencing)
	Background lecture series: Molecular evolution
Week 2	Hands-on: Introduction to the Bash command line environment
	Hands-on: Introduction to base-calling and quality assessment of sequencing data
	Background lecture series: Molecular evolution
**Part 2—Genome Assembly, Annotation, and Downstream Analyses**	
Week 3	Hands-on: Genome assembly (long-read assembly) and polishing (long-read/short-read)
	Hands-on: Transcriptome assembly
	Background lecture series: Molecular evolution
Week 4	Hands-on: Assembly quality assessment (assembly statistics, BUSCO)
	Hands-on: Scaffolding to chromosome level using Hi-C data
	Hands-on: Genome annotation (repetitive elements, genes)
	Background lecture series: Molecular evolution
	Seminar: Current research examples in vertebrate genomics
Week 5	Hands-on: Introduction to comparative analyses, e.g., methods of phylogenetic tree reconstruction or population genomics
	Background lecture series: Molecular evolution
Week 6	Theory: Introduction to scientific writing
	Theory: Overview of the scientific publishing process (from manuscript writing to publication)
	Hands-on: Manuscript writing
	General quality assessment session

Detailed information can be found in Supplementary Material 1.

The selection of a species for a MinION-based genome assembly course should be based on a few characteristics: (i) the availability of relatively fresh material for the extraction of hmwDNA; (ii) prior testing of its ability to be sequenced on a MinION because some taxa cannot effectively be sequenced on a MinION, probably owing to the presence of biological molecules in the DNA extraction that interfere with the sequencing process; (iii) genome size because this dictates how much data and computational resources are needed for a successful assembly; (iv) the interest of the community in the species; and (v) availability of short-read data for polishing or chromosome-level scaffolding.

In these courses, we have focused on teleost fish genomes for which we have established hmwDNA extraction and MinION sequencing protocols [9]. Among vertebrates, many teleost fish species have relatively small genomes (∼400–700 Mb), and low coverage (20–30×) of long-read data is usually sufficient to generate high-quality genome assemblies for these. We highly recommend using available databases such as the Animal Genome Size Database [http://genomesize.com/] to look up genome size estimations for a target species during course planning. Alternatively, short-read data or flow cytometry can be used to estimate genome sizes. There are a variety of genome assemblies available online (databases: NCBI Genome [www.ncbi.nlm.nih.gov/genome], DNA Zoo [www.dnazoo.org], GigaDB [www.gigadb.org], etc.) that are based on short-read libraries. These would benefit from more continuous assemblies with long-read data to allow for more in-depth analyses such as on genome architecture evolution or speciation. Because NCBI and other genome databases require all the accompanying raw read data to be deposited on their SRA database (https://www.ncbi.nlm.nih.gov/sra), these reads could also be used for genome polishing during the course. Even though individual read error rates (5–25%, reviewed in [2, 3]) for the MinION have decreased over recent years, it is still recommended to polish the resulting genome assemblies using highly accurate (0.1–1% error rate, reviewed in [2]) short-read data. To produce chromosome-level genome assemblies, so-called proximity-ligation sequencing data are needed. Several companies offer kits to generate these. However, the library construction is complicated and usually requires 2 full days, so we do not recommend generating these during the course. Alternatively, public data platforms such as DNA Zoo offer useful resources for proximity-ligation data. Furthermore, these databases include numerous species for which high-quality, continuous assemblies are unavailable.

The development of time- and resource-effective genome assembly tools, such as wtdbg2 [10], allows students to generate genome assemblies of vertebrate genomes within hours, even on small servers. Depending on the genome size and amount of read data, it might even be possible to assemble the genomes on a desktop computer or laptop. It is advantageous to teach the students first how to run these tools on a subset of the data and then to have them process the complete data in smaller groups. This way, relatively time-intensive steps can be processed overnight, and the results checked and discussed with the students the following day. Subsequently, they can be introduced to post-assembly steps such as repeat or gene annotation.

The generation and assembly of genomic data as part of university courses also makes it possible to involve students in the publishing process of peer-reviewed scientific publications. To achieve this, we allocated time to include scientific writing and publishing in the curriculum. The students were given the task to draft a genome announcement paper under the supervision of the course trainers, which was then submitted to bioRxiv (https://www.biorxiv.org/) (see, e.g., [9]) and a peer-reviewed scientific journal. This way, students are involved in every step of the process, from generating the genomic data to assembly and annotation and to publishing the scientific article. They will not only learn how to write and publish scientific manuscripts but are also involved in publishing scientific peer-reviewed articles very early in their scientific careers. This keeps the motivation high because they have the opportunity to work on new data and obtain meaningful results compared to the analysis of simplified teaching datasets, often used as classroom examples. Collaborations with different research groups can help to find scientifically interesting species to sequence, which will ensure that publication of the genome assembly and annotation is of general interest for the research community.

## Conclusions

Here we show that recent advances in portable sequencing technology, ever-decreasing sequencing costs, the development of computationally efficient tools, and the increasing availability of publicly accessible read data can be used for practical teaching of genome assembly and genomics within the frame of university master courses. Selection of species with smaller genomes or more reliance on available data may also enable universities in low-income areas and countries to organize genome assembly courses. Practical training that focuses on newly sequenced or improved genome assemblies will further enable students to gain experience publishing scientific studies early on in their careers.

## Additional Files

Supplementary Material 1: Course structure and teaching goals

giaa058_GIGA-D-20-00088_Original_Submission

giaa058_GIGA-D-20-00088_Revision_1

giaa058_Response_to_Reviewer_Comments_Original_Submission

giaa058_Reviewer_1_Report_Original_Submission Bernie Pope, Ph.D. -- 4/14/2020 Reviewed

giaa058_Supplemental_File

## Abbreviations

BUSCO: Benchmarking Universal Single-Copy Orthologs; hmwDNA: high molecular weight DNA; Mb: megabase pairs; NCBI: National Center for Biotechnology Information; SRA: Sequence Read Archive; USB: Universal Serial Bus.

## References

[1] International Human Genome Sequencing Consortium. Initial sequencing and analysis of the human genome. Nature. 2001;409:860–921. 11237011 10.1038/35057062

[2] Goodwin S, McPherson JD, McCombie WR Coming of age: ten years of next-generation sequencing technologies. Nat Rev Genet. 2016;17:333–51. 27184599 10.1038/nrg.2016.49 PMC10373632

[3] Krehenwinkel H, Pomerantz A, Prost S Genetic biomonitoring and biodiversity assessment using portable sequencing technologies: current uses and future directions. Genes. 2019;10:858. 10.3390/genes10110858 PMC6895800 31671909

[4] Jain M, Koren S, Miga KH, et al. Nanopore sequencing and assembly of a human genome with ultra-long reads. Nat Biotechnol. 2018;36:338–45. 29431738 10.1038/nbt.4060 PMC5889714

[5] Jain M, Olsen HE, Paten B, et al. The Oxford Nanopore MinION: delivery of nanopore sequencing to the genomics community. Genome Biol. 2016;17:239. 27887629 10.1186/s13059-016-1103-0 PMC5124260

[6] Salazar AN, Nobrega FL, Anyansi C, et al. An educational guide for nanopore sequencing in the classroom. PLoS Comput Biol. 2020;16:e1007314. 31971941 10.1371/journal.pcbi.1007314 PMC6977714

[7] Zaaijer S, Columbia University Ubiquitous Genomics 2015 class, Erlich Y. Using mobile sequencers in an academic classroom. eLife. 2016;5:e14258. 27054412 10.7554/eLife.14258 PMC4869913

[8] Watsa M, Erkenswick GA, Pomerantz A, et al. Portable sequencing as a teaching tool in conservation and biodiversity research. PLoS Biol. 2020;18(4):e3000667. 32298256 10.1371/journal.pbio.3000667 PMC7188297

[9] Prost S, Petersen M, Grethlein M, et al. Improving the chromosome-level genome assembly of the Siamese fighting fish (*Betta splendens*) in a university master's course. G3: Genes, Genomes, Genetics. 2020, 10.1534/g3.120.401205. PMC7341155 32385046

[10] Ruan J, Li H Fast and accurate long-read assembly with wtdbg2. Nat Methods. 2020;17:155–8. 31819265 10.1038/s41592-019-0669-3 PMC7004874

