# Supplementary material for: Education in the genomics era: Generating high-quality genome assemblies in university courses
Source: Gigascience. 2020 Jun 3;9(6):giaa058. doi: 10.1093/gigascience/giaa058 (PMC7268781; doi:10.1093/gigascience/giaa058)
Supplement: giaa058_GIGA-D-20-00088_Original_Submission [file giaa058_giga-d-20-00088_original_submission.pdf]

GigaScience

Recent advances in sequencing technologies and the increasing amount of publicly available data enable the generation of high-quality genome assemblies in university courses.

--Manuscript Draft--

**Manuscript Number:**	GIGA-D-20-00088
**Full Title:**	Recent advances in sequencing technologies and the increasing amount of publicly available data enable the generation of high-quality genome assemblies in university courses.
**Article Type:**	Commentary
**Funding Information:**	
**Abstract:**	Recent advances in genome sequencing technologies have greatly simplified the generation of genome data, and reduced the costs for genome assemblies, even for complex genomes like that of vertebrates. This 'genomic revolution' calls for more practical genomic courses at universities to prepare students for the increasing importance of genomic data used in virtually all fields of biological and medical research. Low-cost third-generation sequencing technology along with publicly available data, can be used to teach students how to process genomic data, assemble full chromosome-level genomes and publish the results in peer-reviewed scientific journals, or scientific preprint servers. Here we outline experiences gained during two six-week long Master's level courses and discuss practical aspects and considerations for teaching hands-on genome assembly courses.
**Corresponding Author:**	Stefan Prost<br>Senckenberg<br>Frankfurt, GERMANY
**Corresponding Author Secondary Information:**	
**Corresponding Author's Institution:**	Senckenberg
**Corresponding Author's Secondary Institution:**	
**First Author:**	Stefan Prost
**First Author Secondary Information:**	
**Order of Authors:**	Stefan Prost
	Sven Winter
	Jordi De Raad
	Raphael Coimbra
	Magnus Wolf
	Maria Nilsson-Janke
	Malte Petersen
	Deepak Kumar Gupta
	Tilman Schell
	Fritjof Lammers
	Axel Janke
**Order of Authors Secondary Information:**	
**Additional Information:**	
**Question**	**Response**

Powered by Editorial Manager® and ProduXion Manager® from Aries Systems Corporation

Are you submitting this manuscript to a special series or article collection?	No
**Experimental design and statistics**<br><br>Full details of the experimental design and statistical methods used should be given in the Methods section, as detailed in our Minimum Standards Reporting Checklist. Information essential to interpreting the data presented should be made available in the figure legends.<br><br>Have you included all the information requested in your manuscript?	Yes
**Resources**<br><br>A description of all resources used, including antibodies, cell lines, animals and software tools, with enough information to allow them to be uniquely identified, should be included in the Methods section. Authors are strongly encouraged to cite Research Resource Identifiers (RRIDs) for antibodies, model organisms and tools, where possible.<br><br>Have you included the information requested as detailed in our Minimum Standards Reporting Checklist?	Yes
**Availability of data and materials**<br><br>All datasets and code on which the conclusions of the paper rely must be either included in your submission or deposited in publicly available repositories (where available and ethically appropriate), referencing such data using a unique identifier in the references and in the "Availability of Data and Materials" section of your manuscript.<br><br>Have you have met the above requirement as detailed in our Minimum	Yes

Powered by Editorial Manager® and ProduXion Manager® from Aries Systems Corporation

Standards Reporting Checklist?	

Click here to download Manuscript commentary_genomics_v11.docx

Recent advances in sequencing technologies and the increasing amount of publicly available data enable the generation of high-quality genome assemblies in university courses.

Stefan Prost[1,2]*, Sven Winter[3,4] *, Jordi De Raad[1,3], Raphael Coimbra[3,4], Magnus Wolf[3,4], Maria Nilsson-Janke[1,4], Malte Petersen[1], Deepak K. Gupta[1], Tilman Schell[1], Fritjof Lammers[1,3,4] and Axel Janke[1,3,4]

[1]LOEWE-Centre for Translational Biodiversity Genomics, Senckenberg Nature Research Society, Frankfurt, Germany
[2]South African National Biodiversity Institute, National Zoological Garden, Pretoria, South Africa
[3]Institute for Ecology, Evolution and Diversity, Goethe University, Frankfurt, Germany
[4]Senckenberg Biodiversity and Climate Research Centre, Frankfurt, Germany
* Contributed equally.
Correspondence: stefan.prost@senckenberg.de, sven.winter@senckenberg.de

Abstract
Recent advances in genome sequencing technologies have greatly simplified the generation of genome data, and reduced the costs for genome assemblies, even for complex genomes like that of vertebrates. This 'genomic revolution' calls for more practical genomic courses at universities to prepare students for the increasing importance of genomic data used in virtually all fields of biological and medical research. Low-cost third-generation sequencing technology along with publicly available data, can be used to teach students how to process genomic data, assemble full chromosome-level genomes and publish the results in peer-reviewed scientific journals, or scientific preprint servers. Here we outline experiences gained during two six-week long Master's level courses and discuss practical aspects and considerations for teaching hands-on genome assembly courses.

Keywords
Genome assembly, MinION, Oxford Nanopore Technologies, Teaching, University education

Background

The number of published genome assemblies has increased exponentially since the publication of the human genome in 2001 [1,2]. Back then, large international consortia and vast amounts of funding were required to complete these tasks. Today even small research groups can generate high-quality genome assemblies up to full chromosome-level. An important step in this progression was the advent of 'third-generation' sequencing with the release of the Pacific Biosciences' (PacBio) and Oxford Nanopore Technologies' (ONT) sequencing platforms. These technologies perform real-time sequencing of long, single DNA molecules and require no amplification to increase the sequencing detection signal strength [3]. The inclusion of these technologies has drastically improved the quality of generated genome assemblies and substantially decreased costs, thus enabling even small research groups to realise high-quality genome assemblies. The ongoing 'genomic revolution' increased the need for practical university courses on genome assembly and genomics in general.

One of these third-generation sequencing platforms is ONT's MinION. It is an USB-drive-sized sequencer that has gained in popularity over the last five years due to its potential to generate very long reads, its relatively low costs, and its portability [4,5]. Sequencing is performed by measuring ionic current changes when a single-stranded DNA molecule passes through a 'nanopore' in the device's biological membrane [6]. Its portability, ease of use and relatively low costs, make it an ideal and effective teaching tool in classroom settings [7,8], as well as in the field [9]. Studies on the educational use of the MinION device have mainly focused on methods such as DNA- or metabarcoding, or genome sequencing of bacteria or bacteriophages. These approaches have the advantage that they are easy to conduct, do not require large servers for the data processing and are relatively in-expensive. However, today, the combination of (a) nanopore-based sequencing along with (b) new efficient bioinformatic assembly pipelines and (c) (publicly available) short-read data offer the possibility to generate chromosome-level assemblies, even of complex vertebrate genomes, as part of university courses.

Here we report our experience and provide practical aspects of teaching hands-on vertebrate genome assembly courses on the master's level.

Main Text

Over the last two years, we have taught two six-week Master's level courses focusing on the assembly and analysis of vertebrate genomes, and the required theoretical and biological background. In these courses, students gained practical experience in extracting high molecular weight DNA (hmwDNA), preparing sequencing libraries and subsequently sequenced the genomic DNA on the MinION device. These data, in combination with either publicly available or *a priori* generated short-read data, were then used to generate highly continuous vertebrate genome assemblies in the second part of the course.

The selection of a species for a MinION based genome assembly course should be based on a few characteristics: 1) the availability of relatively fresh material for the extraction of hmwDNA, 2) prior testing of its ability to be sequenced on a MinION, as some taxa cannot effectively be sequenced on a MinION, probably due to the presence of biological molecules in the DNA extraction that interfere with the sequencing process, 3) genome size, as this dictates how much data and computational resources are needed for a successful assembly, 4) the interest of the community in the species, and 5) availability of short-read data for polishing or chromosome-level scaffolding.

In our courses, we have focused on teleost fish genomes for which we have established hmwDNA extraction and MinION sequencing protocols [10]. Among vertebrates, many teleost fish species have relatively small genomes (about 400 – 700Mb), and low coverage (20-30x) of long-read data is usually sufficient to generate high-quality genome assemblies for these. We highly recommend using available databases such as www.genomesize.com to look up genome size estimations for a target species when planning the course. Alternatively, short-read data or flow cytometry can be used to estimate genome sizes. There are a variety of genome assemblies available online (databases: www.ncbi.nlm.nih.gov/genome, www.dnazoo.org, www.gigadb.org, etc.) that are based on short-read libraries. These would benefit from more continuous assemblies with long-read data to allow for more in-depth analyses such as on genome architecture evolution or speciation. As NCBI and other genome databases require all the accompanying raw read data to be deposited on their Sequence Read Archive (SRA) database [11], these reads could also be used for genome

polishing during the course. Even though individual read error rates (5–25% [12,13]) for the MinION decreased over the last years, it is still recommended to polish resulting genome assemblies using highly accurate (0.1–1% error rate [14]) short-read data. To produce chromosome-level genome assemblies, so-called proximity-ligation sequencing data are needed. Several companies offer kits to generate these. However, the library construction is complicated and usually requires two full days, so we do not recommend generating these during the course. Alternatively, public data platforms such as www.dnazoo.org offer useful resources for proximity-ligation data. Furthermore, these databases include numerous species for which high-quality, continuous assemblies are unavailable.

The development of time and resource-effective genome assembly tools, such as WTDBG2 [15] allows students to generate genome assemblies of vertebrate genomes within hours, even on small servers. Depending on the genome size and amount of read data, it might even be possible to assemble the genomes on a desktop computer or laptop. It is advantageous to teach the students first how to run these tools on a subset of the data and then have them process the complete data in smaller groups. This way, relatively time-intensive steps can be processed overnight, and the results checked and discussed with the students the following day. Subsequently, they can be introduced to post-assembly steps such as repeat or gene annotation.

The generation and assembly of genomic data as part of university courses also makes it possible to involve students in the publishing process of peer-reviewed scientific publications. To achieve this, we allocated time to include scientific writing and publishing in the curriculum. The students were given the task to draft a genome announcement paper under the supervision of the course trainers, which was then submitted to www.bioRxiv.org (see for example [10]) and a peer-reviewed scientific journal. This way, students are involved in every step of the process, from generating the genomic data, to assembly and annotation, and to publishing the scientific manuscript. This way, students cannot only learn how to write and publish scientific manuscripts but are also involved in publishing scientific peer-reviewed articles very early in their scientific careers. This way, students were highly motivated by the course scheme, because they had the opportunity to work on new data and obtain meaningful results compared to the analysis of simplified mock datasets, often used as classroom examples.

Conclusions

Here we show that recent advances in portable sequencing technology, ever-decreasing sequencing costs, the development of computationally efficient tools, and the increasing availability of publicly accessible read data can be used for practical teaching of genome assembly and genomics within the frame of a university master courses. Selection of species with smaller genomes or a more reliance on available data may also enable universities in low-income areas and countries to organize genome assembly courses. Practical training that focuses on newly sequenced or improved genome assemblies will further enable students to gain experience publishing scientific studies early on in their career.

References

1. Venter JC, Adams MD, Myers EW, Li PW, Mural RJ, Sutton GG, et al. The Sequence of the Human Genome. Science. American Association for the Advancement of Science; 2001;291:1304–51.

2. International Human Genome Sequencing Consortium. Initial sequencing and analysis of the human genome. Nature. Nature Publishing Group; 2001;409:860–921.

3. Goodwin S, McPherson JD, McCombie WR. Coming of age: ten years of next-generation sequencing technologies. Nat Rev Genet. Nature Publishing Group; 2016;17:333–51.

4. Krehenwinkel H, Pomerantz A, Prost S. Genetic Biomonitoring and Biodiversity Assessment Using Portable Sequencing Technologies: Current Uses and Future Directions. Genes. 2019;10:858.

5. Jain M, Koren S, Miga KH, Quick J, Rand AC, Sasani TA, et al. Nanopore sequencing and assembly of a human genome with ultra-long reads. Nat Biotechnol. Nature Publishing Group; 2018;36:338–45.

6. Jain M, Olsen HE, Paten B, Akeson M. The Oxford Nanopore MinION: delivery of nanopore sequencing to the genomics community. Genome Biol. 2016;17:239.

7. Salazar AN, Nobrega FL, Anyansi C, Aparicio-Maldonado C, Costa AR, Haagsma AC, et al. An educational guide for nanopore sequencing in the classroom. PLOS Computational Biology. Public Library of Science; 2020;16:e1007314.

8. Zaaijer S, Columbia University Ubiquitous Genomics 2015 class, Erlich Y. Using mobile sequencers in an academic classroom. Shailes S, editor. eLife. eLife Sciences Publications, Ltd; 2016;5:e14258.

9. Watsa M, Erkenswick GA, Pomerantz A, Prost S. Genomics in the jungle: using portable sequencing as a teaching tool in field courses. bioRxiv. 2019;581728.

10. Prost S, Petersen M, Grethlein M, Hahn SJ, Kuschik-Maczollek N, Olesiuk ME, et al. Improving the chromosome-level genome assembly of the Siamese fighting fish (Betta splendens) in a university Master's course. bioRxiv. 2020;2020.03.06.981332.

11. Kodama Y, Shumway M, Leinonen R. The sequence read archive: explosive growth of sequencing data. Nucleic Acids Res. Oxford Academic; 2012;40:D54–6.

12. Weirather JL, de Cesare M, Wang Y, Piazza P, Sebastiano V, Wang X-J, et al. Comprehensive comparison of Pacific Biosciences and Oxford Nanopore Technologies and their applications to transcriptome analysis. F1000Res [Internet]. 2017 [cited 2020 Feb 18];6. Available from: https://www.ncbi.nlm.nih.gov/pmc/articles/PMC5553090/

13. Wick RR, Judd LM, Holt KE. Deepbinner: Demultiplexing barcoded Oxford Nanopore reads with deep convolutional neural networks. PLoS Comput Biol [Internet]. 2018 [cited 2020 Feb 18];14. Available from: https://www.ncbi.nlm.nih.gov/pmc/articles/PMC6245502/

14. Manley LJ, Ma D, Levine SS. Monitoring Error Rates In Illumina Sequencing. J Biomol Tech. 2016;27:125–8.

15. Ruan J, Li H. Fast and accurate long-read assembly with wtdbg2. Nat Methods. Nature Publishing Group; 2020;17:155–8.
