# Supplementary material for: Education in the genomics era: Generating high-quality genome assemblies in university courses
Source: Gigascience. 2020 Jun 3;9(6):giaa058. doi: 10.1093/gigascience/giaa058 (PMC7268781; doi:10.1093/gigascience/giaa058)
Supplement: giaa058_GIGA-D-20-00088_Revision_1 [file giaa058_giga-d-20-00088_revision_1.pdf]

GigaScience

Education in the genomics era: Generating high-quality genome assemblies in university courses.

--Manuscript Draft--

**Manuscript Number:**	GIGA-D-20-00088R1
**Full Title:**	Education in the genomics era: Generating high-quality genome assemblies in university courses.
**Article Type:**	Commentary
**Funding Information:**	
**Abstract:**	p.p1 {margin: 0.0px 0.0px 0.0px 0.0px; text-align: justify; font: 11.0px 'Times New Roman'; color: #000000; -webkit-text-stroke: #000000} span.s1 {font-kerning: none} Recent advances in genome sequencing technologies have greatly simplified the generation of genome data, and reduced the costs for genome assemblies, even for complex genomes like that of vertebrates. This 'genomic revolution' calls for more practical oriented genomic courses at universities to prepare students for the increasing importance of genomic data used in virtually all fields of biological and medical research. Low-cost third-generation sequencing technology along with publicly available data, can be used to teach students how to process genomic data, assemble full chromosome-level genomes and publish the results in peer-reviewed scientific journals, or scientific preprint servers. Here we outline experiences gained from two Master's level courses and discuss practical aspects and considerations for teaching hands-on genome assembly courses.
**Corresponding Author:**	Stefan Prost<br>Senckenberg<br>Frankfurt, GERMANY
**Corresponding Author Secondary Information:**	
**Corresponding Author's Institution:**	Senckenberg
**Corresponding Author's Secondary Institution:**	
**First Author:**	Stefan Prost
**First Author Secondary Information:**	
**Order of Authors:**	Stefan Prost
	Sven Winter
	Jordi De Raad
	Raphael Coimbra
	Magnus Wolf
	Maria Nilsson
	Malte Petersen
	Deepak Kumar Gupta
	Tilman Schell
	Fritjof Lammers
	Axel Janke
**Order of Authors Secondary Information:**	
**Response to Reviewers:**	see submitted response letter
**Additional Information:**	

Powered by Editorial Manager® and ProduXion Manager® from Aries Systems Corporation

Question	Response
Are you submitting this manuscript to a special series or article collection?	No
**Experimental design and statistics**<br><br>Full details of the experimental design and statistical methods used should be given in the Methods section, as detailed in our Minimum Standards Reporting Checklist. Information essential to interpreting the data presented should be made available in the figure legends.<br><br>Have you included all the information requested in your manuscript?	Yes
**Resources**<br><br>A description of all resources used, including antibodies, cell lines, animals and software tools, with enough information to allow them to be uniquely identified, should be included in the Methods section. Authors are strongly encouraged to cite Research Resource Identifiers (RRIDs) for antibodies, model organisms and tools, where possible.<br><br>Have you included the information requested as detailed in our Minimum Standards Reporting Checklist?	Yes
**Availability of data and materials**<br><br>All datasets and code on which the conclusions of the paper rely must be either included in your submission or deposited in publicly available repositories (where available and ethically appropriate), referencing such data using a unique identifier in the references and in the "Availability of Data and Materials" section of your manuscript.	Yes

Powered by Editorial Manager® and ProduXion Manager® from Aries Systems Corporation

Have you have met the above requirement as detailed in our Minimum Standards Reporting Checklist?	

Manuscript

Click here to access/download;Manuscript;commentary_genomics_Prost_forP

Education in the genomics era:
Generating high-quality genome assemblies in university courses.

Stefan Prost[1,2]*, Sven Winter[3,4] *, Jordi De Raad[1,3], Raphael Coimbra[3,4], Magnus Wolf[3,4], Maria Nilsson[1,4], Malte Petersen[1], Deepak K. Gupta[1], Tilman Schell[1], Fritjof Lammers[1,3,4] and Axel Janke[1,3,4]

[1]LOEWE-Centre for Translational Biodiversity Genomics, Senckenberg Nature Research Society, Frankfurt, Germany
[2]South African National Biodiversity Institute, National Zoological Garden, Pretoria, South Africa
[3]Institute for Ecology, Evolution and Diversity, Goethe University, Frankfurt, Germany
[4]Senckenberg Biodiversity and Climate Research Centre, Frankfurt, Germany
* Contributed equally.
Correspondence: stefan.prost@senckenberg.de, sven.winter@senckenberg.de

Abstract
Recent advances in genome sequencing technologies have greatly simplified the generation of genome data, and reduced the costs for genome assemblies, even for complex genomes like that of vertebrates. This 'genomic revolution' calls for more practical oriented genomic courses at universities to prepare students for the increasing importance of genomic data used in virtually all fields of biological and medical research. Low-cost third-generation sequencing technology along with publicly available data, can be used to teach students how to process genomic data, assemble full chromosome-level genomes and publish the results in peer-reviewed scientific journals, or scientific preprint servers. Here we outline experiences gained from two Master's level courses and discuss practical aspects and considerations for teaching hands-on genome assembly courses.

Keywords
Genome assembly, MinION, Oxford Nanopore Technologies, Teaching, University education

Background

The number of published genome assemblies has increased exponentially since the publication of the human genome in 2001 [1]. Back then, large international consortia and vast amounts of funding were required to complete this task. Today even small research groups can generate high-quality genome assemblies up to full chromosome-level. An important step in this progression was the advent of 'third-generation' sequencing with the release of Pacific Biosciences' (PacBio) and Oxford Nanopore Technologies' (ONT) sequencing platforms. These technologies perform real-time sequencing of long, single DNA molecules and require no amplification to increase the sequencing detection signal strength [2]. The inclusion of these technologies has drastically improved the quality of generated genome assemblies and substantially decreased costs, thus enabling even small research groups to realise high-quality genome assemblies. The ongoing 'genomic revolution' increased the need for practical university courses on genome assembly and genomics in general.

One of these third-generation sequencing platforms is ONT's MinION. It is an USB-drive-sized sequencer that has gained in popularity over the last five years due to its potential to generate very long reads, its relatively low costs, and its portability [3,4]. Sequencing is performed by measuring ionic current changes when a single-stranded DNA molecule passes through a 'nanopore' in the device's biological membrane [5]. Its portability, ease of use and relatively low costs, make it an ideal and effective teaching tool in classroom settings [6,7], as well as in the field [8]. Studies on the educational use of the MinION device have mainly focused on methods such as DNA- or metabarcoding, or genome sequencing of bacteria or bacteriophages. These approaches have the advantage that they are easy to conduct, do not require large servers for the data processing and are relatively in-expensive. However, today, the combination of (a) nanopore-based sequencing along with (b) new efficient bioinformatic assembly pipelines and (c) (publicly available) short-read data offer the possibility to generate chromosome-level assemblies, even of complex vertebrate genomes, as part of university courses.

Here we report our experience and provide practical aspects of teaching hands-on vertebrate genome assembly courses on the master's level.

Main Text

Over the last two years, we have taught two Master's level courses, each six weeks long, focusing on the assembly and analysis of vertebrate genomes, and the required theoretical and biological background. In these courses, students gained practical experience in extracting high molecular weight DNA (hmwDNA), preparing sequencing libraries and subsequently sequenced the genomic DNA on the MinION device in the first part of the course (see Table 1 and Supplementary Material 1). These data, in combination with either publicly available or previously generated short-read data, were then used to generate highly continuous vertebrate genome assemblies in the second part of the course (see Table 1 and Supplementary Material 1). The inclusion of dedicated sessions on the basics of laboratory work and bioinformatics enables the active participation of students from a variety of disciplines (such as biology or bioinformatics) even without prior knowledge in these fields. We recommend to keep the number of students lower than 15 to enable direct interactions. This is important especially for students that have no or little bioinformatics or laboratory experience, and might otherwise struggle to keep up with the course.

The selection of a species for a MinION based genome assembly course should be based on a few characteristics: 1) the availability of relatively fresh material for the extraction of hmwDNA, 2) prior testing of its ability to be sequenced on a MinION, as some taxa cannot effectively be sequenced on a MinION, probably due to the presence of biological molecules in the DNA extraction that interfere with the sequencing process, 3) genome size, as this dictates how much data and computational resources are needed for a successful assembly, 4) the interest of the community in the species, and 5) availability of short-read data for polishing or chromosome-level scaffolding.

In these courses, we have focused on teleost fish genomes for which we have established hmwDNA extraction and MinION sequencing protocols [9]. Among vertebrates, many teleost fish species have relatively small genomes (about 400 – 700Mb), and low coverage (20-30x) of long-read data is usually sufficient to generate high-quality genome assemblies for these. We highly recommend using available databases such as www.genomesize.com to look up genome size estimations for a target species when planning the course.

Alternatively, short-read data or flow cytometry can be used to estimate genome sizes. There are a variety of genome assemblies available online (databases: www.ncbi.nlm.nih.gov/genome, www.dnazoo.org, www.gigadb.org, etc.) that are based on short-read libraries. These would benefit from more continuous assemblies with long-read data to allow for more in-depth analyses such as on genome architecture evolution or speciation. As NCBI and other genome databases require all the accompanying raw read data to be deposited on their Sequence Read Archive (SRA) database (https://www.ncbi.nlm.nih.gov/sra), these reads could also be used for genome polishing during the course. Even though individual read error rates (5–25%, reviewed in [2,3]) for the MinION decreased over the last years, it is still recommended to polish resulting genome assemblies using highly accurate (0.1–1% error rate, reviewed in [2]) short-read data. To produce chromosome-level genome assemblies, so-called proximity-ligation sequencing data are needed. Several companies offer kits to generate these. However, the library construction is complicated and usually requires two full days, so we do not recommend generating these during the course. Alternatively, public data platforms such as www.dnazoo.org offer useful resources for proximity-ligation data. Furthermore, these databases include numerous species for which high-quality, continuous assemblies are unavailable.

The development of time and resource-effective genome assembly tools, such as WTDBG2 [10] allows students to generate genome assemblies of vertebrate genomes within hours, even on small servers. Depending on the genome size and amount of read data, it might even be possible to assemble the genomes on a desktop computer or laptop. It is advantageous to teach the students first how to run these tools on a subset of the data and then have them process the complete data in smaller groups. This way, relatively time-intensive steps can be processed overnight, and the results checked and discussed with the students the following day. Subsequently, they can be introduced to post-assembly steps such as repeat or gene annotation.

The generation and assembly of genomic data as part of university courses also makes it possible to involve students in the publishing process of peer-reviewed scientific publications. To achieve this, we allocated time to include scientific writing and publishing in the curriculum. The students were given the task to draft a genome announcement paper under the supervision of the course trainers, which was then submitted to www.bioRxiv.org (see for example [9]) and a peer-reviewed scientific journal. This way, students are involved in every step of the process, from generating the genomic data, to assembly and annotation, and to publishing the scientific manuscript. They will not only learn how to write and publish scientific manuscripts but are also involved in publishing scientific peer-reviewed articles very early in their scientific careers. This keeps the motivation high, because they have the opportunity to work on new data and obtain meaningful results compared to the analysis of simplified teaching datasets, often used as classroom examples. Collaborations with different research groups can help to find scientifically interesting species to sequence, which will ensure that publication of the genome assembly and annotation is of general interest for the research community.

Conclusions

Here we show that recent advances in portable sequencing technology, ever-decreasing sequencing costs, the development of computationally efficient tools, and the increasing availability of publicly accessible

read data can be used for practical teaching of genome assembly and genomics within the frame of a university master courses. Selection of species with smaller genomes or a more reliance on available data may also enable universities in low-income areas and countries to organize genome assembly courses. Practical training that focuses on newly sequenced or improved genome assemblies will further enable students to gain experience publishing scientific studies early on in their career.

References

1. International Human Genome Sequencing Consortium. Initial sequencing and analysis of the human genome. Nature. Nature Publishing Group; 2001;409:860–921.

2. Goodwin S, McPherson JD, McCombie WR. Coming of age: ten years of next-generation sequencing technologies. Nat Rev Genet. Nature Publishing Group; 2016;17:333–51.

3. Krehenwinkel H, Pomerantz A, Prost S. Genetic Biomonitoring and Biodiversity Assessment Using Portable Sequencing Technologies: Current Uses and Future Directions. Genes. 2019;10:858.

4. Jain M, Koren S, Miga KH, Quick J, Rand AC, Sasani TA, et al. Nanopore sequencing and assembly of a human genome with ultra-long reads. Nat Biotechnol. Nature Publishing Group; 2018;36:338–45.

5. Jain M, Olsen HE, Paten B, Akeson M. The Oxford Nanopore MinION: delivery of nanopore sequencing to the genomics community. Genome Biol. 2016;17:239.

6. Salazar AN, Nobrega FL, Anyansi C, Aparicio-Maldonado C, Costa AR, Haagsma AC, et al. An educational guide for nanopore sequencing in the classroom. PLOS Computational Biology. Public Library of Science; 2020;16:e1007314.

7. Zaaijer S, Columbia University Ubiquitous Genomics 2015 class, Erlich Y. Using mobile sequencers in an academic classroom. Shailes S, editor. eLife. eLife Sciences Publications, Ltd; 2016;5:e14258.

8. Watsa M, Erkenswick GA, Pomerantz A, Prost S (2020) Portable sequencing as a teaching tool in conservation and biodiversity research. PLoS Biol 18(4): e3000667. https://doi.org/10.1371/journal.pbio.3000667

9. Prost S, Petersen M, Grethlein M, Hahn SJ, Kuschik-Maczollek N, Olesiuk ME, et al. Improving the chromosome-level genome assembly of the Siamese fighting fish (Betta splendens) in a university Master's course. bioRxiv. 2020;2020.03.06.981332.

10. Ruan J, Li H. Fast and accurate long-read assembly with wtdbg2. Nat Methods. Nature Publishing Group; 2020;17:155–8.

Table
Click here to access/download;Table;commentary_genomics_Prost_revision1

Tables

**Part 1 - Laboratory Processing and Basic Training**		
	Week1	- **Theory:** Introduction to Genome sequencing techniques and analyses<br>- **Hands-on:** Laboratory work (laboratory safety guidelines, DNA Isolation, quality assessment, Nanopore Library Preparation, Sequencing)<br>- **Background lecture series:** Molecular Evolution
	Week 2	- **Hands-on:** Introduction to the bash command line environment<br>- **Hands-on:** Intro to base-calling and quality assessment of sequencing data<br>- **Background lecture series:** Molecular Evolution
**Part 2 - Genome Assembly, Annotation and Downstream Analyses**		
	Week 3	- **Hands-on:** Genome assembly (long-read assembly) and polishing (long-read/short-read)<br>- **Hands-on:** Transcriptome assembly<br>- **Background lecture series:** Molecular Evolution
	Week 4	- **Hands-on:** Assembly Quality assessment (assembly statistics, BUSCO)<br>- **Hands-on:** Scaffolding to chromosome level using Hi-C data<br>- **Hands-on:** Genome annotation (repetitive elements, Genes)<br>- **Background lecture series:** Molecular Evolution<br>- **Seminar:** Current research examples in Vertebrate genomics
	Week 5	- **Hands-on:** Introduction to comparative analyses: e.g., Methods of phylogenetic tree reconstruction or population genomics<br>- **Background lecture series:** Molecular Evolution
	Week 6	- **Theory:** Introduction to scientific writing<br>- **Theory:** Overview of the scientific publishing process (from manuscript writing to publication)<br>- **Hands-on:** Manuscript writing<br>- **General QA Session**

Table 1. Example of a course outline. Detailed information can be found in the Supplementary Material 1.

Click here to access/download
Supplementary Material
commentary_genomics_Prost_revision1_SUPPL.docx

Cover Letter
Click here to access/download;Personal Cover;response_letter_Prost_revision1.docx

Dear Hans et al.,

Please, find our revisions and below our comments to the really helpful review. I hope we have addressed everything to your satisfaction.

All the best,
Stefan

CC: scott@gigasciencejournal.com

GIGA-D-20-00088
Recent advances in sequencing technologies and the increasing amount of publicly available data enable the generation of high-quality genome assemblies in university courses.
Stefan Prost; Sven Winter; Jordi De Raad; Raphael Coimbra; Magnus Wolf; Maria Nilsson-Janke; Malte Petersen; Deepak Kumar Gupta; Tilman Schell; Fritjof Lammers; Axel Janke
GigaScience

Dear Stefan,

Many thanks for submitting your "Commentary" to Gigascience. We feel it is a good contribution for the journal. We have also shown it to an external expert, to get some input from another pair of eyes. Bernie Pope (University of Melbourne) kindly read your commentary and provided some helpful feedback - please see his comments below.

I particularly agree with Bernie that it would be helpful to present a kind of example curriculum, and other info that would make it easier for our readers to take practical hints from the commentary, if they want to do something similar at their own institutions.

We have included a basic example curriculum as a table and a much more detailed discussion of it as a supplementary. We thought a supplementary would be better than a box, since it is quite a bit of text. Please, let us know if that is ok.

One technical point to keep in mind during the revision is that the "Commentary" article type has restrictions regarding length and number of references. The length of the present version is about right, it should not get substantially longer - but you can add infoboxes or figures to include some of the information suggested by the reviewers (text in "info boxes" and figures does not count to the total word number). If you wish to share teaching resources, as suggested by the referee, I think we could also add them as supplemental material.

See our comments above.

One strict limitation for "commentaries" is that they can not have more than 10 references - do you think you can cut some (for example, maybe one reference to the human genome project is enough, and maybe some others are redundant or can be replaced by URLs in the text?)

We reduced the references to 10.

I cc Scott, our executive editor, in case he has any additional comments.

Once you have made the necessary corrections, please submit a revised manuscript online at:

https://www.editorialmanager.com/giga/

If you have forgotten your username or password please use the "Send Login Details" link to get your login information. For security reasons, your password will be reset.

The due date for submitting the revised version of your article is 14 Jul 2020.

We look forward to receiving your revised manuscript soon.

Best wishes,

Hans

Hans Zauner
GigaScience
www.gigasciencejournal.com

Reviewer reports:
Reviewer #1: This manuscript provides a short commentary on the authors' findings from running a genome assembly course at Masters level, based on 2 years of experience. They argue that recent advances in long read sequencing technology (particularly MinION) and efficient assembly tools have made genome assembly practical in a teaching setting.

The commentary provides some useful pointers to resources that may be helpful for other educators who are interested in doing something similar.

While I think that this paper provides some interesting insights, I feel that it is missing a few important details that would be important for the target audience, and I would encourage these to be considered before publication:

- Who are the target students of the genome assembly course? From the paper it appears that the students are engaged in some laboratory processes (e.g. DNA extraction). Therefore, one assumes they will need to have laboratory skills. The students will also perform some amount of computational data analysis (e.g. running genome assembly and annotation software). Therefore, they will also need to have computational skills. I think it would be beneficial for the paper to outline the prerequisites for students admitted to such a course. Would it be suitable for a purely bioinformatics student, or a purely biology student? Could the laboratory component be avoided for students who do not have the requisite skills?

We have included a short section on how we structured the course so that no prior skills are necessary.

- It would be useful for the readers to have a more detailed curriculum outline. It is not clear from the paper how long each of the parts of the course will take. The authors mention that the overall teaching takes place in two six-week Master's level courses. However, it is not clear how the material is split across the two courses. The authors also talk about "the second part of the course", but it is not clear what this refers to. Is it the second of the two courses? As part of this it might also be useful to explain how assessment is done in this kind of course. What are the expected learning outcomes?

We have included a curriculum and a discussion thereof. We also made the partitioning more clear in the text.

- Have the authors collected feedback from the students, and if so (and students have agreed to this being published), what can be learnt from their feedback?

We did not collect feedback on the structure per se, but talked with the students after the course, so that we can improve it the next year. The current presented version is the outcome of the first round of student discussion.

- Do the authors have any teaching materials that may be shared with other educators, in order to help them adopt a similar kind of subject?

Not yet, unfortunately. But we are looking into a more formal way to establish online teaching material for similar courses.

- Typically how many students would be enrolled in such a course at any one time? Are there constraints on student numbers due to resource limitations (e.g. access to sequencing machines etc)?

We have included that info.

- It is interesting that the authors have included a component of manuscript preparation into the subject. This is a clever idea and is both teaching the students a useful skill and also potentially motivating. However, I wonder how scalable this will be. Presumably there is a limit to the number of different genomes that can be used for such a course? If the manuscripts are being submitted to a preprint archive or to journals, they presumably must satisfy novelty criteria. How can an educator ensure this is sustainable over a longer period of time without becoming burdensome?

Yes, we think that is possible. It is true that it gets much harder to publish genome assemblies. However, in order to avoid having issues with publishing, we decided to create full chromosome-level assemblies, and (which is included now) we have collaborations with other research sections to sequence a species that will be part of a research project either way. Also, it is always possible to submit genome announcements just to preprint servers like bioRxiv - though a peer-reviewed publication would always be preferable.

- Have the authors considered using a computational workflow system such as Galaxy (or equivalent) to help the students access computational resources?

We have not thought about that, as neither one has really ever used it. Also, the field is moving very rapidly and we often need to exchange tools between courses as newer and faster tools develop. We simply do not know if workflows such as Galaxy develop as quickly.

Very minor comments:

- I would suggest replacing "a priori" with an English equivalent, to improve the readability of the paper.

We change this to “prior”.

We thank you and Bernie Pope for the valuable comments!

All the best,
Stefan Prost
