# Supplementary material for: Education in the genomics era: Generating high-quality genome assemblies in university courses
Source: Gigascience. 2020 Jun 3;9(6):giaa058. doi: 10.1093/gigascience/giaa058 (PMC7268781; doi:10.1093/gigascience/giaa058)
Supplement: giaa058_Supplemental_File [file giaa058_supplemental_file.docx]

**Supplementary - Course structure and teaching goals**

The course is divided into two parts. The first one focuses on the laboratory processing and the basics needed for the subsequent data analysis that is taught in the second part of the course (see also main manuscript **Table 1**). We run regular lectures and have textbook and paper seminars during the course to provide the students with the necessary theoretical background on genetics and genomes, and to give them an overview of current research in these areas.

**Part 1 - Laboratory Processing and Basic Training**

The **first week** starts with a general course outline to provide the students with a framework of the mile-stones, followed by a two day detailed introduction to genome assembly, sequencing techniques and analyses. Materials covered are later reiterated in short lectures throughout the practical sessions to ensure repetition. Next, we teach students the required laboratory skills, including laboratory safety, and procedures such as hmwDNA isolation, quality assessment, MinION library preparation and subsequent sequencing. To ensure that students without or with limited prior experience in laboratory techniques can acquire the necessary basic skills, we allocated three days for this part. We restrict the hands-on processing to DNA extraction and MinION library preparation and sequencing. These are relatively basic laboratory procedures. Including Illumina library preparation, especially of Hi-C data, would likely add too much complexity for the students. These data types can be generated upfront during the preparation of the course in addition to a MinION test run. The beforehand generated data can also function as backup to ensure the course can be run, if the laboratory steps during the course fail for any reason.

In the **second week**, we introduce the students over a period of two days to working on a server using the command line to teach them simple bash commands, which are necessary to perform the bioinformatic processing in Part 2. Subsequently, we start the base-calling of the MinION sequencing runs. Here and in Part 2, we first teach the students the basics of each bioinformatic step on a subset of the data, followed by the students working in small groups on the full data. We found group sizes from 2-3 students to be an ideal balance between helping each other and being directly involved.

**Part 2 - Genome Assembly, Annotation and Downstream Analyses**

**Week 3:** We start by looking at the quality of the total read data, followed by genome assembly and polishing. We run different genome assembly tools and discuss the pros and cons of these, and discuss long-read versus short-read polishing strategies. During this week, we also introduce transcriptome assembly and how this process differs from genome assembly. If needed, this week can be spread out over a longer period to allow students more time to familiarize themselves with the necessary concepts and software tools.

In **week 4**, we first cover the topics: genome assembly quality assessment and chromosome-level scaffolding using Hi-C data, followed by genome annotation. Given the complexity of genome annotation, we set aside three days for this.

In **Week 5,** we introduce students to various phylo- and population genomic analyses. This will provide them with an overview of current research on these topics. To this end we included a seminar with individual student presentations on related and current research papers.

In the last week (**week 6**), we cover the basics of scientific writing and the publishing process. This also includes a practical session in which students draft a genome announcement paper based on the results from the course. Even though not part of the course due to time limitations, in the following weeks we involve the students in the full paper writing and publishing process via email and meetings.

**Student Assessment**

The students can be evaluated using different criteria: the quality of the course protocol, their participation, seminar presentations and/or the final exam. To keep them engaged we assigned the highest weight (60% of the grade) to the course protocol. This should be written in the form of a step-by-step manual that can be used for future reference by the students themselves, for example if they continue on with graduate projects that involve genome assembly. In order to test how much the students have understood and studied the materials covered during the course, we put the second highest weight on the final exam (30%). The research paper seminar counts the smallest amount towards the final grade (10%).
