# Supplementary material for: Education in the genomics era: Generating high-quality genome assemblies in university courses
Source: Gigascience. 2020 Jun 3;9(6):giaa058. doi: 10.1093/gigascience/giaa058 (PMC7268781; doi:10.1093/gigascience/giaa058)
Supplement: giaa058_Response_to_Reviewer_Comments_Original_Submission [file giaa058_response_to_reviewer_comments_original_submission.pdf]

Author's Response To Reviewer Comments

Close

see submitted response letter

Close
