# Supplementary material for: Education in the genomics era: Generating high-quality genome assemblies in university courses
Source: Gigascience. 2020 Jun 3;9(6):giaa058. doi: 10.1093/gigascience/giaa058 (PMC7268781; doi:10.1093/gigascience/giaa058)
Supplement: giaa058_Reviewer_1_Report_Original_Submission — Bernie Pope, Ph.D. -- 4/14/2020 Reviewed [file giaa058_reviewer_1_report_original_submission.pdf]

Reviewer Report

Title: Education in the genomics era: Generating high-quality genome assemblies in university courses.

Version: Original Submission **Date:** 4/14/2020

Reviewer name: Bernie Pope, Ph.D.

Reviewer Comments to Author:

This manuscript provides a short commentary on the authors' findings from running a genome assembly course at Masters level, based on 2 years of experience. They argue that recent advances in long read sequencing technology (particularly MinION) and efficient assembly tools have made genome assembly practical in a teaching setting.

The commentary provides some useful pointers to resources that may be helpful for other educators who are interested in doing something similar.

While I think that this paper provides some interesting insights, I feel that it is missing a few important details that would be important for the target audience, and I would encourage these to be considered before publication:

- Who are the target students of the genome assembly course? From the paper it appears that the students are engaged in some laboratory processes (e.g. DNA extraction). Therefore, one assumes they will need to have laboratory skills. The students will also perform some amount of computational data analysis (e.g. running genome assembly and annotation software). Therefore, they will also need to have computational skills. I think it would be beneficial for the paper to outline the prerequisites for students admitted to such a course. Would it be suitable for a purely bioinformatics student, or a purely biology student? Could the laboratory component be avoided for students who do not have the requisite skills?
- It would be useful for the readers to have a more detailed curriculum outline. It is not clear from the paper how long each of the parts of the course will take. The authors mention that the overall teaching takes place in two six-week Master's level courses. However, it is not clear how the material is split across the two courses. The authors also talk about "the second part of the course", but it is not clear what this refers to. Is it the second of the two courses? As part of this it might also be useful to explain how assessment is done in this kind of course. What are the expected learning outcomes?
- Have the authors collected feedback from the students, and if so (and students have agreed to this being published), what can be learnt from their feedback?
- Do the authors have any teaching materials that may be shared with other educators, in order to help them adopt a similar kind of subject?
- Typically how many students would be enrolled in such a course at any one time? Are there constraints on student numbers due to resource limitations (e.g. access to sequencing machines etc)?
- It is interesting that the authors have included a component of manuscript preparation into the subject. This is a clever idea and is both teaching the students a useful skill and also potentially motivating. However, I wonder how scalable this will be. Presumably there is a limit to the number of different genomes that can be used for such a course? If the manuscripts are being submitted to a preprint archive or to journals, they presumably must satisfy novelty criteria. How can an educator

ensure this is sustainable over a longer period of time without becoming burdensome?
- Have the authors considered using a computational workflow system such as Galaxy (or equivalent) to help the students access computational resources?
Very minor comments:
- I would suggest replacing "a priori" with an English equivalent, to improve the readability of the paper.

Level of Interest

Please indicate how interesting you found the manuscript: Choose an item.

Quality of Written English

Please indicate the quality of language in the manuscript: Choose an item.

Declaration of Competing Interests

Please complete a declaration of competing interests, considering the following questions:

- Have you in the past five years received reimbursements, fees, funding, or salary from an organisation that may in any way gain or lose financially from the publication of this manuscript, either now or in the future?
- Do you hold any stocks or shares in an organisation that may in any way gain or lose financially from the publication of this manuscript, either now or in the future?
- Do you hold or are you currently applying for any patents relating to the content of the manuscript?
- Have you received reimbursements, fees, funding, or salary from an organization that holds or has applied for patents relating to the content of the manuscript?
- Do you have any other financial competing interests?
- Do you have any non-financial competing interests in relation to this paper?

If you can answer no to all of the above, write 'I declare that I have no competing interests' below. If your reply is yes to any, please give details below.

I declare that I have no competing interests

I agree to the open peer review policy of the journal. I understand that my name will be included on my report to the authors and, if the manuscript is accepted for publication, my named report including any attachments I upload will be posted on the website along with the authors' responses. I agree for my report to be made available under an Open Access Creative Commons CC-BY license (http://creativecommons.org/licenses/by/4.0/). I understand that any comments which I do not wish to be included in my named report can be included as confidential comments to the editors, which will not be published.

Choose an item.

To further support our reviewers, we have joined with Publons, where you can gain additional credit to further highlight your hard work (see: https://publons.com/journal/530/gigascience). On publication of this paper, your review will be automatically added to Publons, you can then choose whether or not to claim your Publons credit. I understand this statement.

Yes Choose an item.
